# Mind the Gaps: Revisiting the Validity, Consistency, and Scope of the Eating Disorder Examination. A Commentary on Reilly et al. (2025)

**DOI:** 10.1002/eat.24509

**Published:** 2025-07-22

**Authors:** Ricarda Schmidt

**Affiliations:** ^1^ Research Unit Behavioral Medicine, Integrated Research and Treatment Center AdiposityDiseases, Department of Psychosomatic Medicine and Psychotherapy University of Leipzig Medical Center Leipzig Germany; ^2^ German Center for Child and Adolescent Health (DZKJ), partner Site Leipzig/Dresden Leipzig Germany

**Keywords:** assessment, diagnosis, measurement, psychometric properties

## Abstract

This commentary responds to Reilly et al.'s (2025) forum article and focuses primarily on Area of Focus #2: ensuring group‐specific validity and adaptability of eating disorder assessment tools. Using the Eating Disorder Examination (EDE) as a case example, it is argued that psychometric flexibility must be accompanied by empirical accountability. Specifically, the commentary highlights the importance of testing measurement invariance (MI) to evaluate whether tools like the EDE function equivalently across different populations and time points. This is particularly relevant as the EDE or its self‐report version (EDE‐Q) are increasingly being used in populations for which they were not originally designed, for example, individuals with avoidant/restrictive food intake disorder or people of diverse gender identities. Additionally, the commentary discusses the need for harmonization between different versions of the instrument (EDE vs. EDE‐Q), and calls for greater transparency in reporting and applying scoring conventions. A further consideration is the consistency of application across raters and research contexts, suggesting that interrater reliability should be examined more systematically across sites. Drawing on the metaphor of a long‐serving but evolving vehicle, the commentary argues that modernization is necessary, but must not come at the cost of clinical depth or training relevance. Knowing how to drive remains essential, even when upgrading the vehicle.

## 1 Introduction

In their timely and thoughtful forum article, Reilly et al. (2025) call for a field‐wide rethinking of how eating disorders (ED) are assessed. Specifically, the authors focused on the most widely used tool for assessing EDs and their associated psychopathology, the Eating Disorder Examination (EDE; Cooper and Fairburn 1987). That the EDE, now in its 17th version, has endured for decades, even as diagnostic criteria, treatment paradigms, and societal understandings of EDs have shifted, is both a testament to its utility and institutional embeddedness. However, the intervals between updates have grown longer, with the last revision dating back to 2014, which may be related to the retirement of Christopher Fairburn, the EDE's lead author and driving force behind its development. His departure may have left a vacuum in leadership that now invites collective responsibility. Reilly et al.'s forum paper can be seen as an attempt to fill that gap and to initiate a broader, collaborative renewal process. Drawing on past critiques of the EDE (e.g., Thomas et al. 2014), Reilly et al. (2025) identified three areas in need of field‐wide consensus: (1) aligning tool selection with the specific goals and context of assessment (e.g., screening vs. diagnosis); (2) ensuring that tools are valid and adaptable for use across diverse demographic and clinical groups; and (3) improving the transparency and standardization of how tools are implemented, adapted, and reported in research and practice. These Areas of Focus converge on a shared concern: ensuring that the EDE is both clinically meaningful and psychometrically robust in diverse contexts.

## 2 The EDE in Transition

To frame the scope of this commentary, imagine the EDE as a long‐serving public bus. It was originally designed to run a specific route—serving passengers with anorexia nervosa (AN) and bulimia nervosa (BN). Over time, however, it has increasingly been used to travel along different routes than initially intended (e.g., applied to individuals with avoidant/restrictive food intake disorder [ARFID]), and to transport a more diverse group of passengers, many of whom have long been present in the population but were not previously acknowledged or adequately represented (e.g., individuals from gender‐minority groups). The EDE has even begun to drive itself in its self‐report version, the EDE‐Questionnaire (EDE‐Q). Meanwhile, some important destinations—such as muscularity‐oriented concerns—are not yet part of the mapped network. And there is concern that different drivers (i.e., interviewers) may interpret instructions differently or take inconsistent paths. Despite these expansions and uncertainties, we often assume that the EDE delivers all passengers to the same destination, in the same way. This commentary explores whether that assumption holds—whether the EDE functions equivalently across diverse clinical or demographic groups, interviewers, and versions. Thus, this commentary focuses primarily on Reilly et al.'s Area of Focus #2—group‐specific validity and adaptability. However, it also touches on related concerns discussed in Area of Focus #1 and #3, such as the use of the EDE outside its original diagnostic context, and inconsistencies between different versions of the tool (e.g., the EDE and EDE‐Q) and different interviewers.

## 3 Evaluating Measurement Invariance

One of the central challenges highlighted in Reilly et al.'s Area of Focus #2—ensuring group‐specific validity and adaptability—is determining whether an instrument functions equivalently across different populations and contexts. This issue, however, is not new. A decade earlier, Thomas et al. (2014) raised critical concerns about the EDE's psychometric assumptions, including the lack of empirical support for its factor structure, conceptual overlap between subscales, and limited applicability across diagnostic and gender groups. While the authors did not specifically address MI, their critique directly aligns with the kinds of questions MI analyses are designed to answer. MI provides a structured, empirical method for testing whether observed score differences reflect true variation or measurement bias, making it a critical, though underused, tool for addressing long‐standing limitations of the EDE. While MI relates most directly to Area of Focus #2, it also has implications for Area #1 (tool selection) and Area #3 (reporting), as it offers concrete evidence on when and for whom a tool yields valid comparisons.

Reilly et al. (2025) propose that we retrofit the EDE vehicle: widen the doors for broader access, update the route map to reflect current destinations, and ensure that every part of the vehicle—from signage to seating—serves its function clearly and fairly. A key part of this modernization is assessing whether the vehicle performs equally well for all passengers. In psychometric terms, MI analyses allow us to test whether key constructs, such as “restraint” or “shape concern”, carry the same meaning across populations and time points (Putnick and Bornstein 2016). This includes testing whether the factor structure holds across groups (configural invariance), whether item loadings are equal (metric invariance), and whether item intercepts are comparable (scalar invariance). Or, metaphorically speaking: the EDE may appear to follow the same route for everyone, but in fact may take different paths (i.e., noninvariant factor structure), stop at different stations (i.e., noninvariant item intercepts), or respond differently to who is on board (i.e., unequal item loadings across groups).

Although interest in MI has grown over the past decade, there remains a lack of robust evidence for MI not only for the EDE or EDE‐Q, but ED assessment tools in general. Most studies evaluating MI of the EDE or EDE‐Q have focused on alternative model structures rather than testing the original four‐factor solution, consistently demonstrating invariance in latent structure, item loadings, and intercepts between men and women and across racial and ethnic groups (with some exceptions). Only limited consideration has been given to other variables including age, gender minority status, profession, language, geographic region, diet, or weight status. While these findings at least challenge the critique that the EDE was developed only for a selected social group (individuals identifying as White and cisgender female), they also highlight the near absence of MI analyses based on the original EDE factor model and—most importantly—across different ED diagnoses and time points. Yet comparisons across these categories are highly common in ED research.

Regarding cross‐group validity, a large‐scale study by Laskowski et al. (2023) who applied Exploratory Graph Analysis to the EDE‐Q demonstrated substantial structural differences between women with AN, BN, and binge‐eating disorder (BED). For example, in the AN group, only 12 of the 22 attitudinal EDE‐Q items could be retained due to item instability. The study by Laskowski et al. (2023) indicated that the EDE‐Q's original model may not generalize equally well across ED diagnoses. Nevertheless, it provided evidence that core aspects of ED psychopathology, such as restraint and body dissatisfaction, can still be assessed across diagnostic groups, albeit with fewer items and dimensions than initially proposed.

Importantly, MI is not a panacea for the theoretical limitations of the EDE, but it offers a crucial means of evaluating whether those assumptions hold up across contexts. However, some limitations of MI must be acknowledged: its application is sensitive to sample size and group heterogeneity, and it relies on a reasonably well‐fitting and theoretically grounded measurement model. These limitations underscore the need for careful use and interpretation. As part of a broader modernization effort, MI can help evaluate whether new or modified versions of the EDE function equivalently across groups and contexts, thus guiding evidence‐based refinements. Note that MI can inform assessment selection (Area of Focus #1) by indicating when certain subscales or items may not function equivalently in specific populations, and it can contribute to Area of Focus #3 by encouraging more transparent reporting of psychometric properties across groups. In sum, MI may serve as a bridge between conceptual inclusivity and empirical accountability.

## 4 Scoring Consistency Across Research Groups

Beyond testing MI, another critical and underexplored issue is interrater reliability across research contexts. While MI analyses evaluate whether the structure and meaning of an instrument's scores are equivalent across groups or time points, they do not capture variability introduced by the rater. Interrater reliability becomes essential to assess whether those applying the instrument do so consistently across settings and samples. This aspect aligns closely with Reilly et al.'s Area of Focus #3, which emphasizes the need for greater transparency and consistency in how the EDE is administered, adapted, and reported across research groups. Reilly et al. (2025) note that training practices and institutional norms may influence the EDE's scoring, raising the question of how consistently the tool is actually applied in practice. In terms of the earlier metaphor: even if the bus itself is structurally sound, the way it is driven may vary. Do all drivers follow the same route, stop at the designated stations, and observe the same speed limits? Multi‐site interrater reliability studies—using blinded assessors rating identical interviews—could reveal systematic deviations between research groups. Such data would inform not only fidelity to the tool but also potential sources of scoring drift across settings. These evaluation efforts would complement psychometric refinement by capturing the lived variability of how the EDE is used across settings.

## 5 Aligning EDE and EDE‐Q Use

In striving for consistency and comparability, it is also essential to acknowledge structural discrepancies between the EDE and the EDE‐Q. Though often used interchangeably, these two formats partly operate under different assumptions—akin to two versions of the same bus model, one driven by a professional driver (the EDE) and one self‐driving (the EDE‐Q). While the former includes clear instructions for when to stop and whom to pick up (e.g., rating dissatisfaction only if shape is perceived as too large), the latter allows for a greater degree of interpretation (because specific rating instructions are lacking), potentially picking up different types of passengers for the same stop. As a result, individuals dissatisfied with their shape due to feeling “too small” (as may occur, for example, in ARFID, men, or in non‐clinical samples) can score identically in the EDE‐Q to those expressing dissatisfaction with shape based on feeling “too large”. Such inconsistencies highlight the need for harmonization and transparent reporting, as emphasized in Reilly et al.'s Area of Focus #3—and they raise important questions about tool selection and context‐specific validity (Area of Focus #1).

## 6 Beyond AN and BN: Limits of Generalization

The EDE was not designed to be all things to all researchers. As Reilly et al. (2025) rightly emphasize, its original purpose was to assess core features of AN and BN from a cognitive‐behavioral perspective, not to capture the clinical presentations of diagnoses such as ARFID. Nevertheless, the EDE and EDE‐Q are increasingly used in ARFID research, including in our own studies. Notably, emerging evidence suggests substantial heterogeneity in EDE or EDE‐Q global scores among individuals with ARFID: while some score above the clinical threshold of 4, many score close to 0. This variation appears to be associated, in part, with weight status—individuals with ARFID and higher versus lower weight tend to report higher levels of ED psychopathology on the EDE or EDE‐Q.

Although such use may yield exploratory insights into traditional ED psychopathology in individuals with ARFID, it also underscores the limits of applying the EDE and EDE‐Q beyond their intended diagnostic scope. This highlights the need for diagnosis‐specific validation (Area of Focus #2) and more careful alignment between assessment tools and their intended purpose (Area of Focus #1).

## 7 Conclusion

The EDE has been a milestone in the field of ED assessment, shaping how we measure ED psychopathology. At the same time, it has served as a training ground for clinical listening and diagnostic reasoning, teaching generations of clinicians and researchers how to navigate the road of ED diagnosis and treatment. Importantly, continued use of the EDE, alongside thoughtful modernization, also helps to preserve essential clinical skills. If diagnostic decisions were to rely solely on automated or technology‐based tools, we risk losing opportunities to train the next generation in nuanced, interaction‐based assessment. Knowing how to drive remains just as vital as improving the vehicle.

As we now consider upgrading the EDE vehicle, we should do so with care. Ensuring MI, assessing scoring consistency, and harmonizing different versions are not peripheral details; they are critical checkpoints on the road to fair, valid, and inclusive assessment.

## Author Contributions

**Ricarda Schmidt:** writing – review and editing, writing – original draft.

## Conflicts of Interest

Ricarda Schmidt, in collaboration with colleagues, has developed a supplementary module to the Eating Disorder Examination for the assessment of avoidant/restrictive food intake disorder. There are no other conflicts of interest.

## Data Availability

Data sharing not applicable to this article as no datasets were generated or analysed during the current study.
